# Supplementary figures and images for: Endothelial nitric oxide synthase (eNOS) S1176 phosphorylation status governs atherosclerotic lesion formation
Source: Front Cardiovasc Med. 2023 Nov 14;10:1279868. doi: 10.3389/fcvm.2023.1279868 (PMC10683645; doi:10.3389/fcvm.2023.1279868)

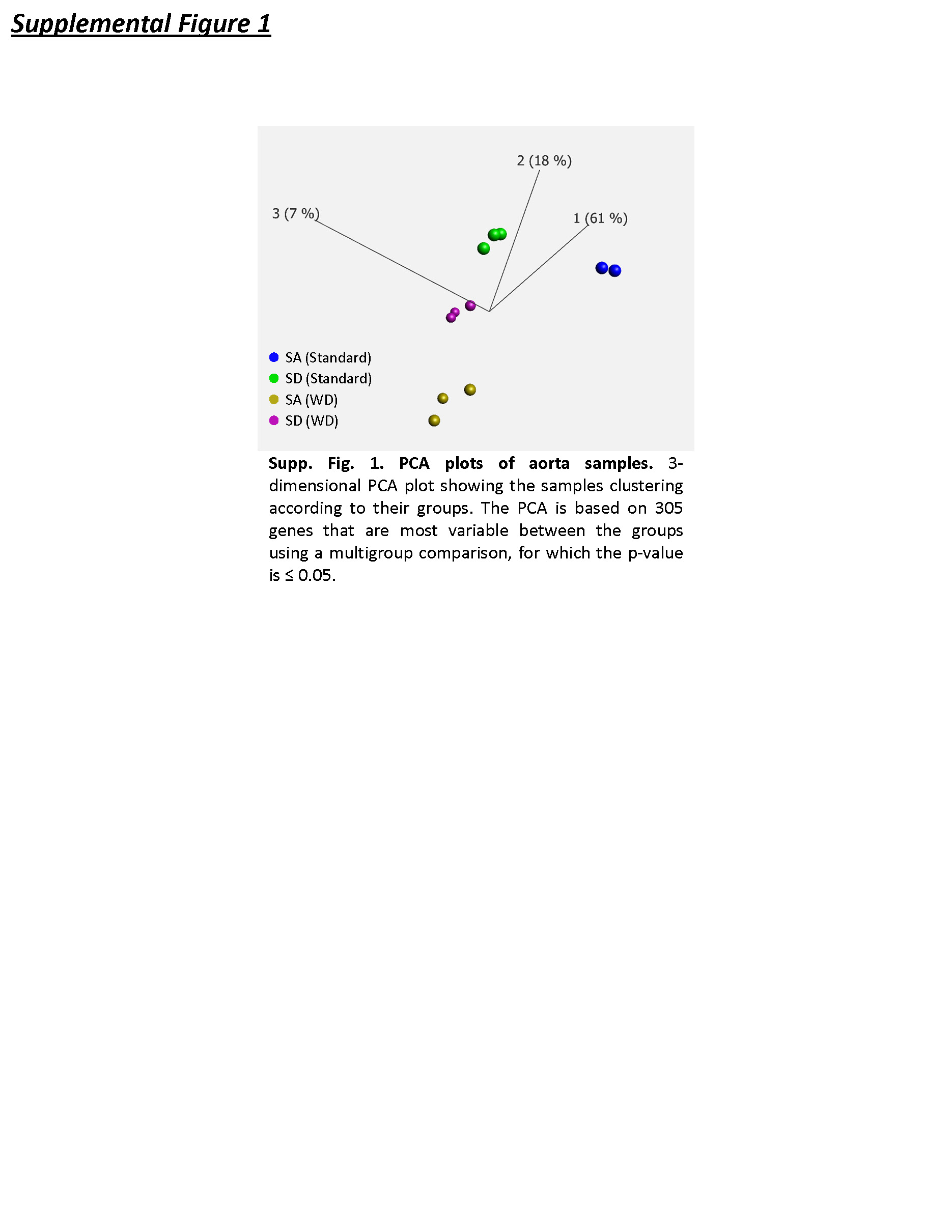

Supplement: Supplementary file 1 [file Image1.jpeg]

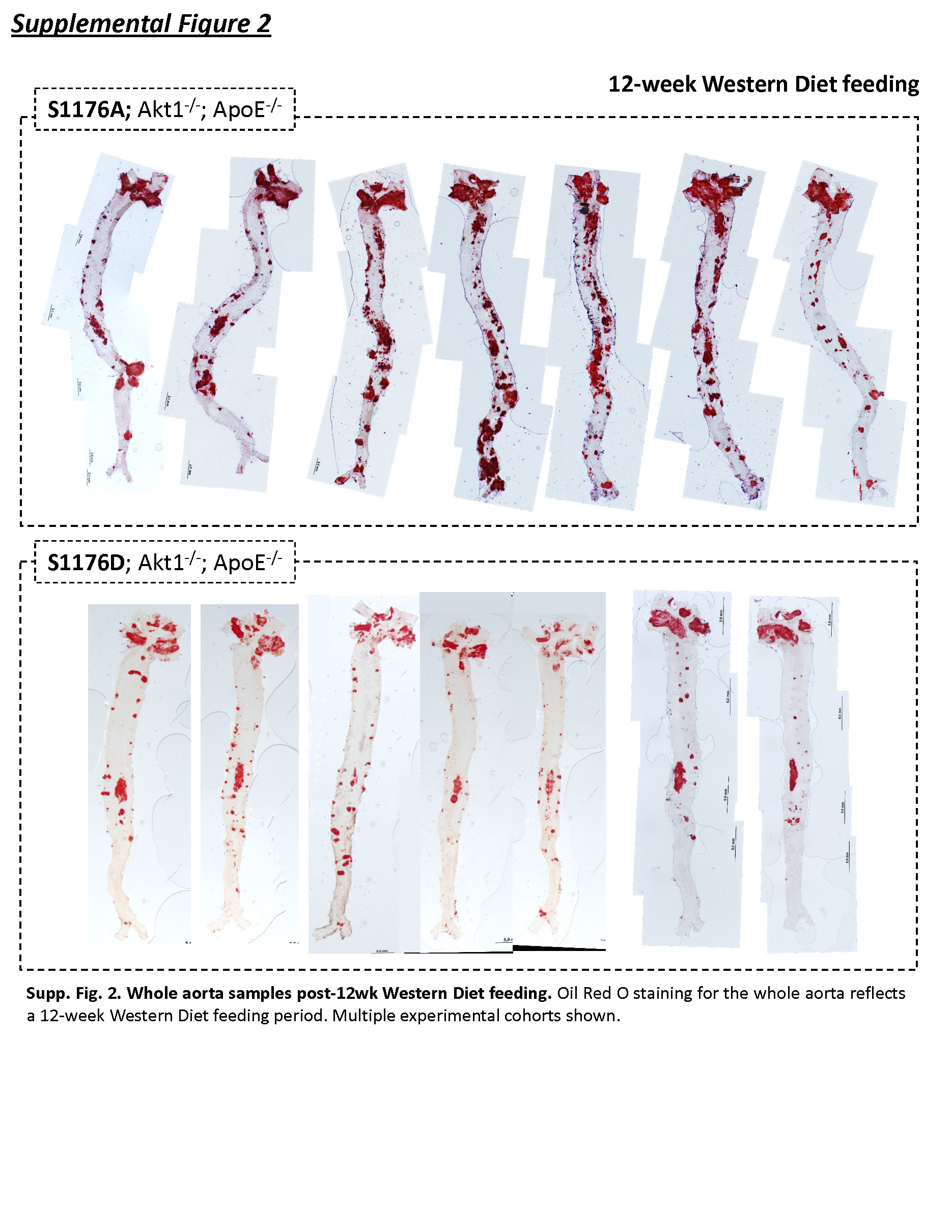

Supplement: Supplementary file 2 [file Image2.jpeg]

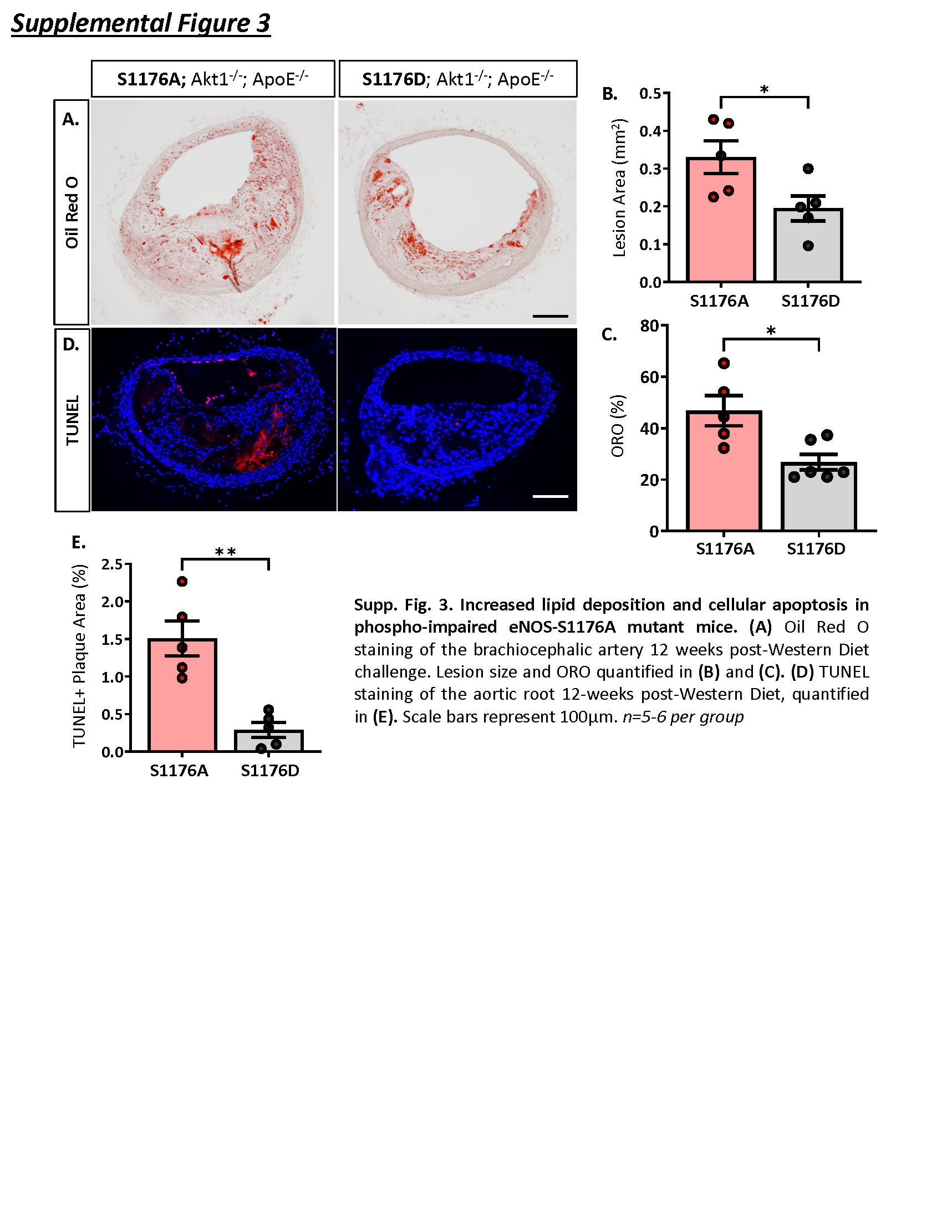

Supplement: Supplementary file 3 [file Image3.jpeg]

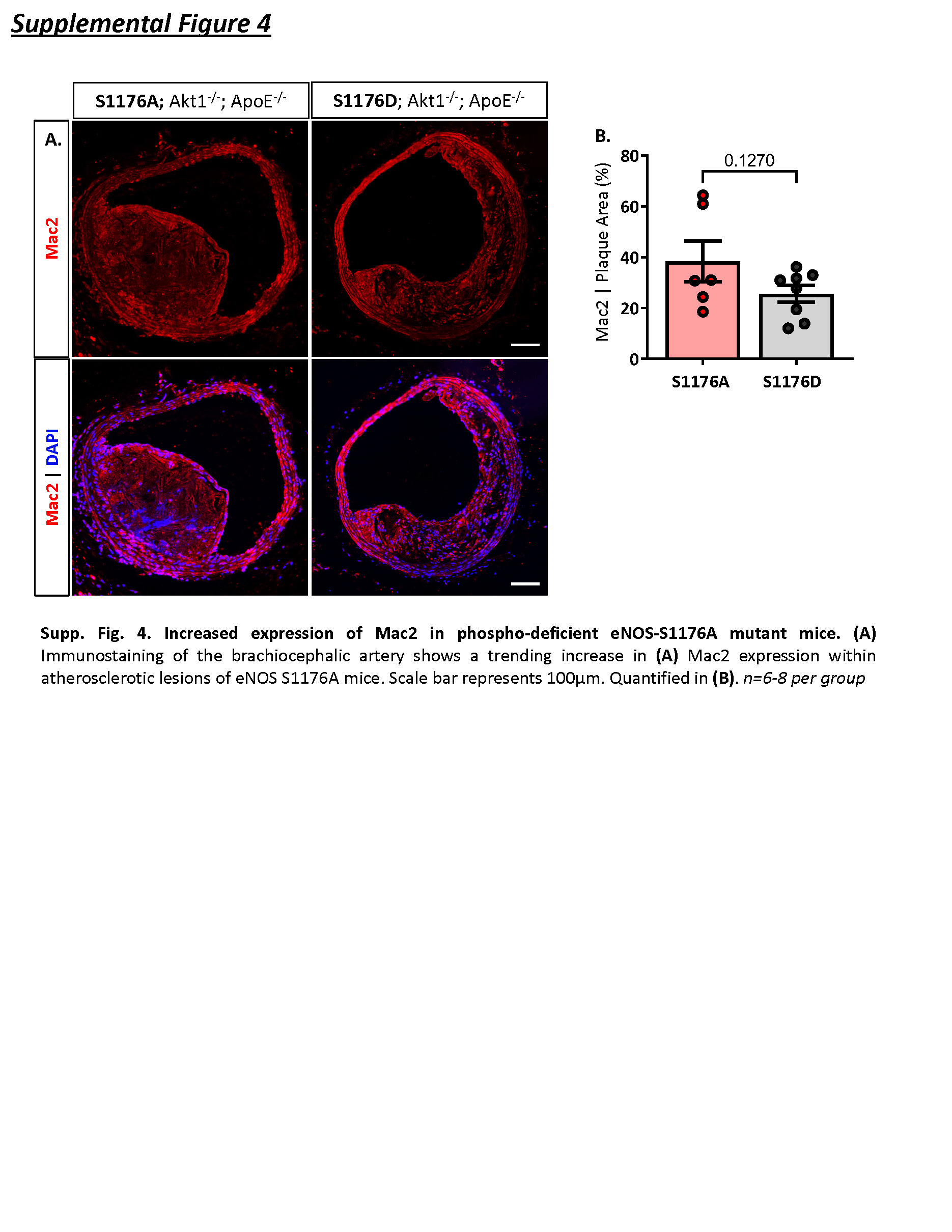

Supplement: Supplementary file 4 [file Image4.jpeg]

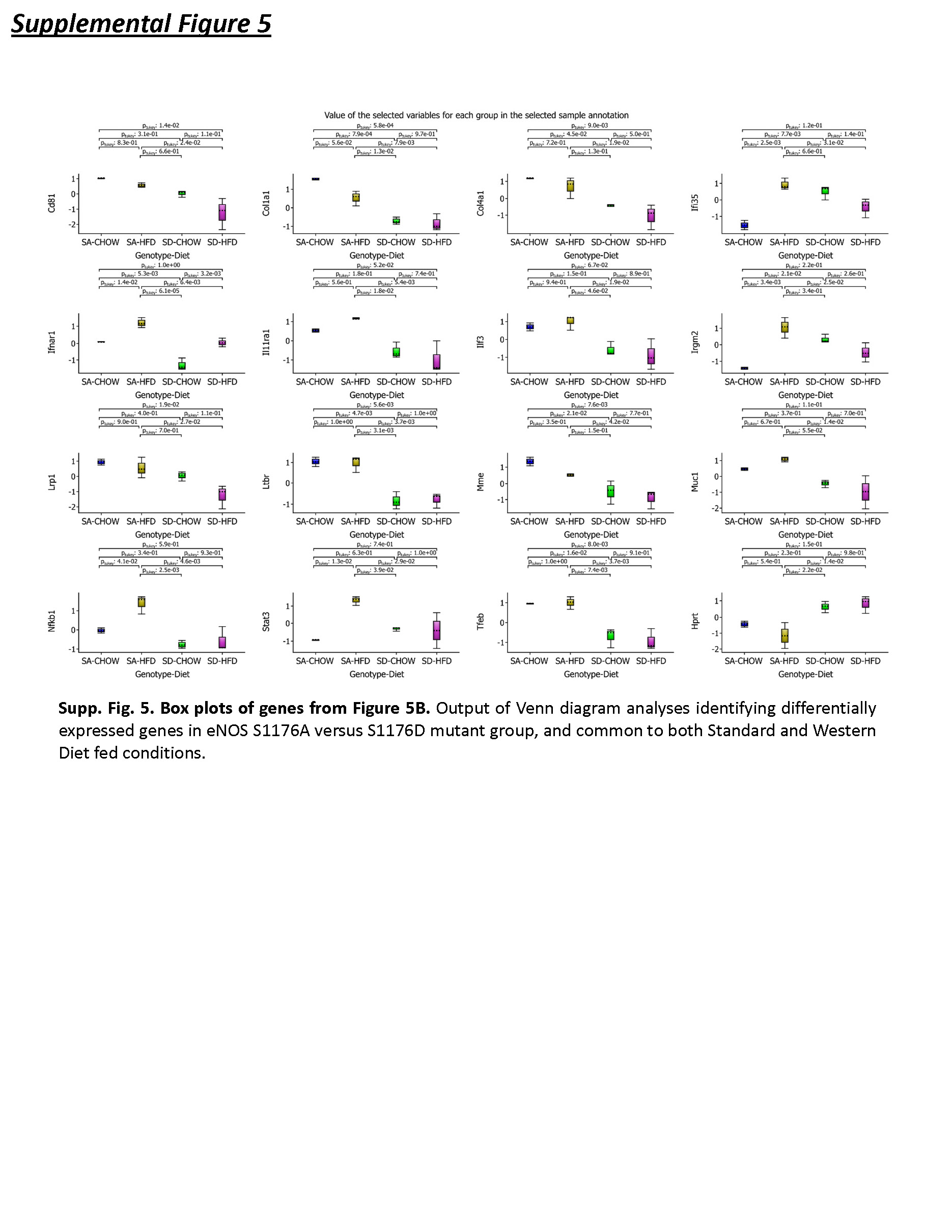

Supplement: Supplementary file 5 [file Image5.jpeg]

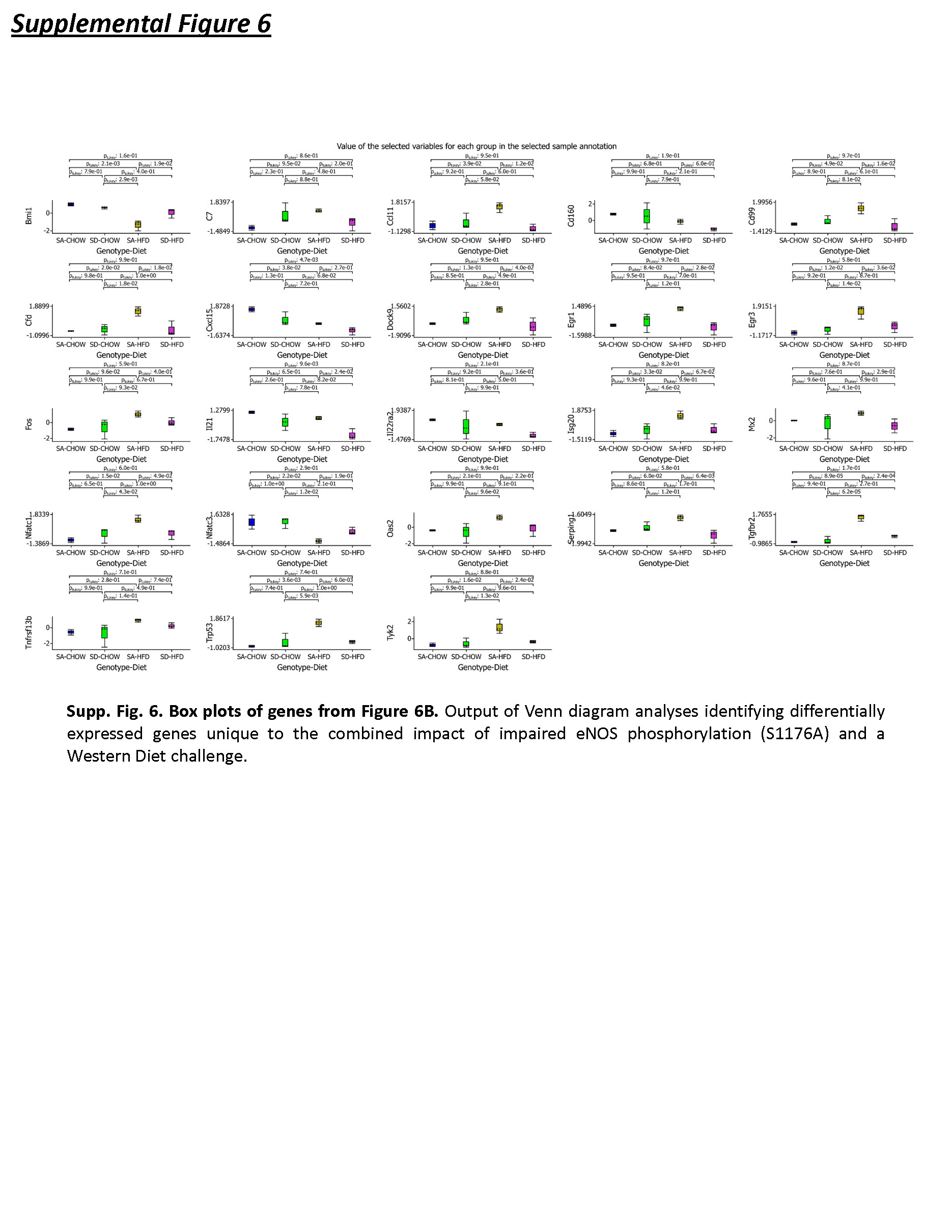

Supplement: Supplementary file 6 [file Image6.jpeg]
